# Effectiveness and Safety of Cryoablation in Patients With Atrial Fibrillation Episodes of <24 h Duration: A Propensity-Matched Analysis

**DOI:** 10.3389/fcvm.2021.724378

**Published:** 2021-10-26

**Authors:** Chunying Jiang, Dongdong Zhao, Kai Tang, Yiqian Wang, Xiang Li, Peng Jia, Yawei Xu, Bing Han

**Affiliations:** ^1^Department of Cardiology, Shanghai Tenth Clinical Medical School of Nanjing Medical University, Shanghai Tenth People's Hospital, Shanghai, China; ^2^Department of Cardiology, The Xuzhou School of Clinical Medicine of Nanjing Medical University, Xuzhou Central Hospital, Xuzhou, China

**Keywords:** cryoablation, paroxysmal atrial fibrillation, pulmonary veins isolation, atrial fibrillation episodes of <24-h duration, recurrence of atrial tachyarrhythmia

## Abstract

**Background:** Paroxysmal atrial fibrillation (AF) is closely related to pathophysiologic processes and clinical outcomes. However, it is uncertain whether cryoablation of pulmonary veins isolation is effective and safe for patients with symptomatic and drug refractory AF episodes of <24-h duration.

**Methods:** The patients were designed into Group A (253 patients with paroxysmal AF episodes of <24-h duration) and Group B (253 patients with paroxysmal AF lasting for 24 h or longer) on a 1:1 basis by identical propensity scores. Mortality, stroke/transient ischemic attack (TIA), and complications relevant to the cryoablation procedure were compared, and recurrence of atrial tachyarrhythmia was analyzed for clinical independent predictors.

**Results:** The rate of atrial tachyarrhythmia recurrence was 21.74% in Group A and 30.04% in Group B, respectively (*P* = 0.042). At 12-month follow-up from the procedure, lower incidences of stroke/TIA endpoint of the patients were observed in Group A compared with Group B by Kaplan–Meier analysis [HR 0.34 (0.13–0.87), *P* = 0.025]. No significant differences in mortality and complications relevant to the cryoablation procedure were observed between Group A and Group B. Moreover, adjusted multivariable Cox regression analysis showed that <24-h paroxysmal AF type (HR 0.644, 95% CI: 0.455–0.913, *P* = 0.014) and left atrium diameter (LAD) (>40 mm) (HR 1.696, 95% CI: 1.046–2.750, *P* = 0.032) were independently associated with the incidence of recurrence of atrial tachyarrhythmia in the study.

**Conclusion:** Our findings indicated that <24-h paroxysmal AF type was obviously associated with an increased success rate of cryoablation and reduced incidence of stroke/TIA during the follow-up period. Therefore, there is superior effectiveness and similar safety in patients with AF episodes of <24-h duration compared with patients with longer paroxysmal AF duration.

## Introduction

Atrial fibrillation (AF), the most common sustained arrhythmia in adults, is associated with an increased risk of morbidity and mortality in the general population (1). A very promising and successful interventional therapy for symptomatic and drug refractory AF is cryoablation of the left atrium (LA), which isolates pulmonary veins (PVs) targeting the initiating triggers inside the PVs in patients with no or minimal structural heart disease (2). Paroxysmal AF, which is defined as episode duration <7 days recommend by contemporary North American and European guidelines, may not reflect pathophysiologic processes or clinical outcomes after catheter ablation (3–5). AF episodes limited to <24 continuous hours are closely related to pathophysiologic processes and clinical outcomes (6).

However, no data to date are available about the effectiveness and safety of cryoablation in patients with AF episodes of <24-h duration. Therefore, the aims of the present study were to evaluate the effectiveness and safety of cryoablation in patients with AF episodes of <24-h duration after adjusting for the confounding variables by propensity-matched analysis.

## Methods

### Study Population

A total of 1,265 consecutive patients presenting with successful cryoballoon-based ablation for symptomatic and drug refractory paroxysmal AF on admission were enrolled in this retrospective and observational study between January 2016 and December 2018 in the Department of Cardiology of Shanghai Tenth People's Hospital. Patients were excluded subsequently with cardiomyopathy, sick sinus syndrome or atrioventricular conduction disturbance, and valvulopathy. Similarly, patients were excluded with age <18 or >80 years, advanced malignancies, severe systemic infections, renal insufficiency, severely reduced liver function, rheumatic and immune disease, thyroid disease, and hematological disorders. Baseline characteristics of all patients were analyzed. Patients were split into the following groups: Group A (patients with paroxysmal AF episodes of <24-h duration) and Group B (patients with paroxysmal AF lasting for 24 h or longer). According to the ethical principles of the Declaration of Helsinki and contemporary North American and European guidelines, written informed consents were obtained from all the subjects (4, 5, 7). This study was granted an exemption from requiring ethics approval by the Ethics Committee of Shanghai Tenth People's Hospital because it was a retrospective observational study.

### Cryoablation Procedure

In accordance with the North American and European guidelines for the management of patients with AF, transesophageal echocardiography was performed on all candidates to exclude the presence of thrombi before the cryoablation procedure (4, 5).

Cryoablation procedures were conducted in the patients following the attainment of consents. A steerable 12-F inner diameter sheath was advanced into the LA through interatrial septum *via* once successful transseptal puncture from the right femoral vein. PV-to-cryoballoon occlusion was assessed with the help of a 50% diluted contrast medium injected into the PV and confirmed by retrograde radiopaque contrast agent retention using fluoroscopic guidance to position the second generation 28-mm cryoballoon catheter. PVs isolation was performed by freezing with a “single-shot” delivery of liquid refrigerant to the balloon, resulting in circumferential and transmural lesions around each pulmonary-vein antrum, and verified by the Achieve mapping catheter during and after each freezing procedure (8). Cryoablation time was 180 s for the isolation of the PVs with the LA (9, 10). In addition, time to isolation was observed by the loss of PV potentials and conduction block during the freezing procedure. In all patients, 30 min after the initial isolation, sustained PVs isolation was demonstrated successfully by the absence of any PV potentials or dissociated PV activity, which failed to induce >30 s rapid atrial arrhythmia by PV stimulation. Just in patients with positive atrial arrhythmia inducibility monitored by the Achieve catheter, additional freezing ablation time of a freezing cycle of 180 s was employed to eliminate residual potentials with a bidirectional conduction block as the endpoint. An activated clotting time of at least 300 s was measured every 30 min, and systemic anticoagulation was maintained by intravenous heparin routinely throughout the cryoablation procedure (11).

Oral antiarrhythmic drugs (AADs) and anticoagulation were routinely administrated and lasted for at least to 3 months according to the guidelines. Subsequently, administration of anticoagulation continued in patients with a ≥2 CHA_2_DS_2_-VASc score on the risk stratification of stroke. Medications were kept based on clinical status of the patients, including beta-blockers, angiotensin-converting enzyme inhibitors (ACEIs) or angiotensin receptor blockers (ARBs), diuretics, and statins.

### Clinical Outcomes and Follow Up

All patients were examined at 1, 3, 6, and 12 months in the outpatient clinic after cryoballoon-based ablation procedure during the follow-up period. ECG recordings and 24-h Holter monitoring were done during every follow-up visit to detect arrhythmias.

The primary composite endpoints of this study after a blanking period of 90 days were as follows: (1) first recurrence of symptomatic or asymptomatic atrial tachyarrhythmia (AF, atrial flutter, or atrial tachycardia) lasting for 30 s or longer by 12-lead ECG or 24-h Holter monitoring in the absence of AADs therapy; (2) appropriate treatment relevant to recurrence of atrial tachyarrhythmia, of AADs prescription (class I or III), or repeat ablation procedure. The major adverse cardiovascular events (MACEs) were defined as secondary endpoints were as follows: all-cause death, cardiac death, stroke, or transient ischemic attack (TIA) from any cause, and complications related to the therapeutic intervention, including phrenic nerve injury (PNI), pericardial tamponade, PV stenosis (PVS), pulmonary embolism (PE), and deep vein thrombosis (DVT).

### Statistical Analysis

A non-parsimonious logistic regression model, namely propensity scores matching, had its confounders adjusted for the likelihood of demographic and clinical variables of each patient enrolled, including age, sex, body mass index, smoking, hypertension, diabetes, previous coronary artery disease (CAD), previous heart failure, history of coronary revascularization, the use of ACEIs, anticoagulants and beta-blockers, left atrium diameter (LAD), left ventricular ejection fraction (LVEF), and CHA_2_DS_2_-VASc score. The patients, from groups of paroxysmal AF episodes of <24-h duration and lasting for 24 h or longer, were matched on a 1:1 basis by using identical propensity scores with a 0.01 standardized caliper width.

All data were analyzed statistically by use of SPSS 26.0. Continuous variables were compared by Student's *t*-test or the Mann–Whitney *U* test appropriately. The differences between categorical values were assessed by the Chi-squared test as appropriate. The survival curves of composite end events were plotted using the Kaplan-Meier method, and significances were explored statistically using the log-rank test. A Cox regression analysis was done to identify the factors associated with the composite endpoint. A *p*-value of <0.05 was considered significant statistically.

## Results

### Baseline Characteristics for Subjects

A total of 1,265 consecutive patients were recruited in the study, including 255 patients with paroxysmal AF episodes of <24-h duration (Group A) and 1,010 patients with paroxysmal AF lasting for 24 h or longer (Group B). Crude baseline clinical characteristics are shown in Table 1. All patients received cryoablation in the study. Among them, patients with paroxysmal AF episodes of <24-h duration were more likely to have younger age (57.50 ± 10.17 vs. 60.39 ± 9.44 years; *P* < 0.001), lower BMI (25.48 ± 3.13 vs. 26.05 ± 2.86 kg/m^2^; *P* = 0.002), a smaller LAD (36.21 ± 4.29 vs. 37.72 ± 4.27 mm; *P* < 0.001), a larger LVEF (55.45 ± 2.22 vs. 54.81 ± 3.25%; *P* = 0.003), and receiving medication of ACEI/ARB (37.25 vs. 26.53%; *P* = 0.001) as compared with patients with paroxysmal AF lasting for 24 h or longer. Overall, 697/1,265 (55.10%) non-propensity-matched patients were on beta-blocker drugs: 122/265 (47.84%) patients of Group A compared to 575/1,100 (56.93%) patients of Group B (*P* = 0.01). Of the whole population, 253/255 (99.22%) cryoablation patients of Group A were matched with 253/1,100 (23.00%) individuals in Group B on a 1:1 basis using identical propensity scores. The baseline characteristics for propensity-matched patients with paroxysmal AF are shown in Table 2 after an adjustment for 15 confounding factors.

**Table 1:** Baseline characteristics for non-propensity-matched patients with paroxysmal AF.

**Characteristics**	**Group A (*n* = 255)**	**Group B (*n* = 1,010)**	***P***
Age, years	57.50 ± 10.17	60.39 ± 9.44	<0.001
Male, *n* (%)	129 (50.59)	463 (45.84)	0.18
BMI, kg/m^2^	25.48 ± 3.13	26.05 ± 2.86	0.002
Smoking, *n* (%)	109 (42.75)	484 (47.92)	0.14
High blood pressure, *n* (%)	190 (74.51)	718 (71.09)	0.31
Diabetes, *n* (%)	60 (23.53)	212 (20.99)	0.39
Prior CAD, *n* (%)	74 (29.02)	308 (30.50)	0.65
Previous heart failure, *n* (%)	15 (5.88)	89 (8.81)	0.16
History of coronary revascularization, *n* (%)	43 (16.86)	199 (19.70)	0.33
LAD, mm	36.21 ± 4.29	37.72 ± 4.27	<0.001
LVEF, %	55.45 ± 2.22	54.81 ± 3.25	0.003
**Medications**			
ACEIs or ARBs, *n* (%)	95 (37.25)	268 (26.53)	0.001
Beta-blockers, *n* (%)	122 (47.84)	575 (56.93)	0.01
Anticoagulants, *n* (%)	111 (43.53)	474 (46.93)	0.36
Mean CHA_2_DS_2_-VASc score	2.10 ± 1.04	2.19 ± 1.05	0.24

*BMI, body mass index; CAD, coronary artery disease; LAD, left atrium diameter; LVEF, left ventricular ejection fraction; ACEIs, angiotensin converting enzyme inhibitors; ARBs, angiotensin receptor blockers*.

**Table 2:** Baseline characteristics for propensitymatched patients with paroxysmal AF.

**Characteristics**	**Group A (*n* = 253)**	**Group B (*n* = 253)**	***P***
Age, years	57.57 ± 10.18	57.47 ± 10.10	0.91
Male, *n* (%)	127 (50.20)	124 (49.01)	0.86
BMI, kg/m^2^	25.51 ± 3.12	25.48 ± 3.17	0.92
Smoking, *n* (%)	109 (43.08)	106 (41.90)	0.86
High blood pressure, *n* (%)	188 (74.30)	186 (73.52)	0.92
Diabetes, *n* (%)	58 (22.92)	56 (22.13)	0.92
Prior CAD, *n* (%)	73 (28.85)	67 (26.48)	0.62
Previous heart failure, *n* (%)	15 (5.93)	18 (7.11)	0.72
History of coronary revascularization, *n* (%)	43 (17.00)	39 (15.42)	0.72
LAD, mm	36.24 ± 4.29	36.33 ± 4.30	0.82
LVEF, %	55.43 ± 2.22	55.40 ± 2.30	0.84
**Medications**			
ACEIs or ARBs, *n* (%)	95 (37.55)	97 (38.33)	0.93
Beta-blockers, *n* (%)	120 (47.43)	119 (47.04)	>0.99
Anticoagulants, *n* (%)	110 (43.48)	106 (41.90)	0.79
Mean CHA_2_DS_2_-VASc score	2.10 ± 1.04	2.07 ± 1.01	0.76

*BMI, body mass index; CAD, coronary artery disease; LAD, left atrium diameter; LVEF, left ventricular ejection fraction; ACEIs, angiotensin converting enzyme inhibitors; ARBs, angiotensin receptor blockers*.

### Follow-Up Analysis

#### Major Adverse Cardiovascular Events

**Figure 1:**
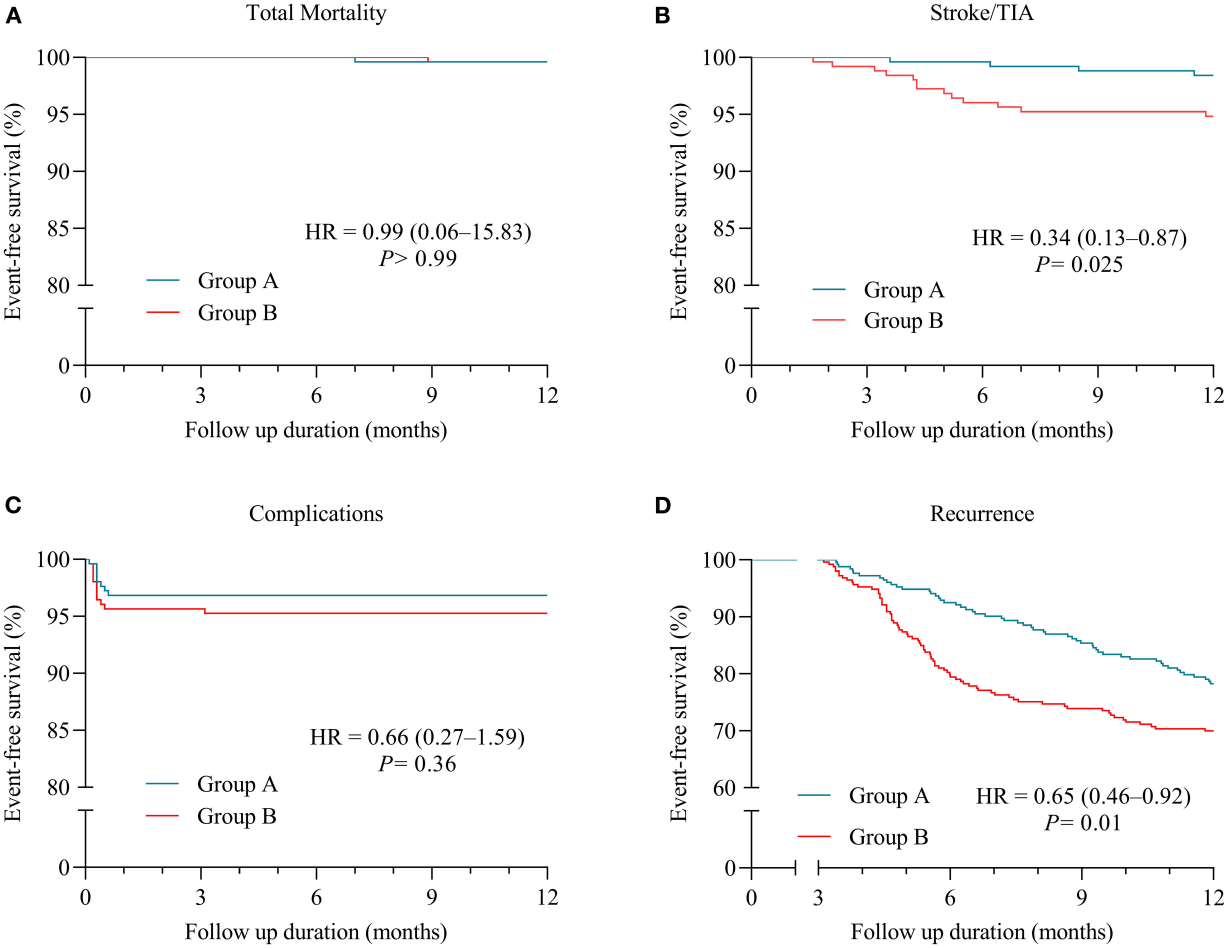
Kaplan-Meier (KM) plots for clinical outcomes in patients with paroxysmal atrial fibrillation (AF) (Group A = patients with AF episodes of <24-h duration; Group B = patients with paroxysmal AF lasting for 24 h or longer). **(A)** KM plot for freedom from total mortality. **(B)** KM plot for freedom from stroke/transient ischemic attack (TIA). **(C)** KM plot for freedom from complications relevant to cryoablation procedure. **(D)** KM plot for freedom from recurrence of AF.

In Table 3, MACEs in patients of Group A are compared with those of propensity-matched patients of Group B during the follow-up period. In the Group A and Group B, the total mortality rates were 0.4 and 0.4% (*P* > 0.99), and cardiovascular death rates were 0.4% and 0 (*P* > 0.99), respectively. Kaplan-Meier survival analysis indicated that the Group A patients had no significant difference of a cumulative incidence of total mortality compared with the Group B patients [HR 0.99 (0.06–15.83), *P* > 0.99] (Figure 1A).

**Table 3:** Mortality and adverse events in propensitymatched patients during the follow-up.

**Parameters**	**Group A (*n* = 253)**	**Group B (*n* = 253)**	***P***
**Mortality**			
Cardiovascular death, *n* (%)	1 (0.40%)	0	>0.99
All-cause death, *n* (%)	1 (0.40%)	1 (0.40%)	>0.99
Stroke or TIA, *n* (%)	4 (1.58%)	13 (5.14%)	0.045
Complications related to cryoablation procedure	9 (3.56%)	13 (5.14%)	0.51
PNI, *n* (%)	7 (2.77%)	9 (3.56%)	0.80
Pericardial tamponade, *n* (%)	1 (0.40%)	2 (0.79%)	>0.99
PVS, *n* (%)	1 (0.40%)	0	>0.99
PE, *n* (%)	0	1 (0.40%)	>0.99
DVT, *n* (%)	0	1 (0.40%)	>0.99

*TIA, transient ischemic attack; PNI, phrenic nerve injury; PVS, pulmonary vein stenosis; PE, pulmonary embolism; DVT, deep vein thrombosis*.

After a follow-up period, stroke/TIA was observed in 1.58% of patients of Group A and 5.14% of Group B (*P* = 0.045) (Table 3). In Figure 1B, Kaplan–Meier curves show lower incidences of the stroke/TIA endpoint of the patients in Group A than Group B [HR 0.34 (0.13–0.87), *P* = 0.025].

All patients received systemic screening for complications related to the therapeutic intervention, which was subsequently observed in nine patients of Group A and 13 patients of Group B (3.56 vs. 5.14%; *P* = 0.51) (Table 3). No significant differences about procedure-related complications, including PNI, pericardial tamponade, PVS, PE, and DVT, were noted between patients from Group A and Group B receiving cryoablation in the Kaplan-Meier survival analysis [HR 0.66 (0.27–1.59), *P* = 0.36] (Figure 1C). PNI occurred in seven (2.77%) patients of Group A and nine (3.56%) patients of Group B in the study population (*P* = 0.80) (Table 3). After a 2-month follow-up, the sniff test showed a total recovery of diaphragmatic palsy in six out of seven (85.71%) patients of Group A and seven out of nine (77.78%) patients of Group B, respectively, and only still mild dyspnea in residual three patients.

#### Recurrence of Atrial Tachyarrhythmia

The recurrence of atrial tachyarrhythmia data was documented from all patients. Kaplan-Meier analysis reveals that lower recurrences of atrial tachyarrhythmia were observed in Group A when compared with Group B [HR 0.65 (0.46–0.92), *P* = 0.01] (Figure 1D). In Table 4, 55 (21.74%) patients in Group A and 76 (30.04%) patients in Group B present with total recurrence of atrial tachyarrhythmia (*P* = 0.042).

**Table 4:** Recurrence of atrial tachyarrhythmia in propensity-matched patients during the follow-up.

**Parameters**	**Group A (*n* = 253)**	**Group B (*n* = 253)**	***P***
Atrial tachyarrhythmia, *n* (%)	55 (21.74)	76 (30.04)	0.042
Atrial tachycardia, *n* (%)	19 (7.51)	35 (13.83)	0.03
Atrial flutter, *n* (%)	18 (7.11)	20 (7.91)	0.87
AF, *n* (%)	18 (7.11)	21 (8.30)	0.74
Early recurrence (4–9 months)	43 (17.00)	71 (28.06)	0.003
Late recurrence (10–12 months)	12 (4.74)	5 (1.98)	0.08
**Appropriate treatments**			
AADs prescription, *n* (%)	11 (4.35)	18 (7.11)	0.25
Direct-current cardioversion, *n* (%)	8 (3.16)	11 (4.35)	0.64
Repeat ablation, *n* (%)	35 (13.83)	47 (18.58)	0.18

*AF, atrial fibrillation; AADs, antiarrhythmic drugs*.

Subgroup analysis is summarized in Table 4 based on atrial tachyarrhythmia types. Ablation patients with <24 h AF had markedly reduced risk of atrial tachyarrhythmia recurrence compared with those with paroxysmal AF episodes sustained more than 24 h (7.51 vs. 13.83%; *P* = 0.03). In patients with AF, the rate of recurrence showed no significant difference between Group A and Group B (7.11 vs. 7.91%; *P* = 0.87, 7.11 vs. 8.30; *P* = 0.74). Lower incidence of early AF recurrence in patients was documented in Group A than Group B (17.00 vs. 28.06%; *P* = 0.003), whereas a similar effect on late AF recurrence for patients was observed from Group A and Group B (4.74 vs. 1.98%; *P* = 0.08). Similarly, appropriate treatments for atrial tachyarrhythmia, including AADs prescription, direct-current cardioversion, and repeat ablation, were statistically similar between the two groups.

#### Predictors of Recurrence of Atrial Tachyarrhythmia

As seen in Table 5, the univariate analysis of the propensity-matched patients shows the incidence of recurrence of atrial tachyarrhythmia during the follow-up period. However, multivariable Cox regression analysis indicated that <24-h paroxysmal AF type (HR 0.644, 95% CI: 0.455–0.913, *P* = 0.014) and LA diameter (>40 mm) (HR 1.696, 95% CI: 1.046–2.750, *P* = 0.032) were independently related to the incidence of recurrence of atrial tachyarrhythmia in the study patients after controlling for confounding factors, including a history of diabetes, prior CAD, and LVEF.

**Table 5:** Cox regression analysis for predictors of recurrence of atrial tachyarrhythmia in propensity-matched patients.

**Variables**	**Univariate analysis**			**Multivariate analysis**		
	**Hazard ratio**	**95% CI**	***P***	**Hazard ratio**	**95% CI**	***P***
AF type (<24 h)	0.656	0.463–0.928	0.017	0.644	0.455–0.913	0.014
Diabetes	0.697	0.445–1.094	0.117	0.846	0.507–1.408	0.518
Prior CAD	0.689	0.454–1.047	0.081	0.735	0.459–1.177	0.200
LAD (>40 mm)	1.956	1.265–3.025	0.003	1.696	1.046–2.750	0.032
LVEF ( ≤ 45%)	3.725	1.523–9.108	0.004	2.554	0.951–6.859	0.063

*AF, atrial fibrillation; CAD, coronary artery disease; LAD, left atrium diameter; LVEF, left ventricular ejection fraction*.

## Discussion

### Main Findings

The main findings of the present study are listed in the following: (1) patients with AF episodes of <24-h duration had lower risks of stroke/TIA and recurrence of atrial tachyarrhythmia than those who had paroxysmal AF lasting for 24 h or longer after a blanking period of 3 months following cryoablation; (2) no significant differences, including total death rate from any cause and complications related to cryoablation procedure, were observed between patients with AF episodes limited to <24 h and those with paroxysmal AF episodes sustained more than 24 h; (3) both <24-h paroxysmal AF type and LA diameter (>40 mm) were independently related to the incidence of recurrence of atrial tachyarrhythmia. The propensity matching analysis was carried out to adjust for confounders of demographic and clinical variables in the evaluation of outcomes of patients. Taken together, these findings confirm the superior effectiveness of cryoablation for patients with AF episodes of <24-h duration compared with patients with longer paroxysmal AF duration *via* a propensity-matched analysis. Furthermore, the safety of cryoablation is similar between the groups.

### Prior Studies

The classification of paroxysmal AF, with episode duration <7 days (3), may not reflect the pathophysiologic process underlying AF or perioperative complications with AF (6). A prior observational and randomized study reported that class IC AADs, such as propafenone and flecainide, were more efficient for the acute conversion of paroxysmal AF or flutter episodes of <24-h duration to sinus rhythm (12). On a secondary analysis of a randomized clinical trial, patients with AF episodes limited to <24 continuous hours had a significantly lower incidence of arrhythmia recurrence following cryoballoon or irrigated radiofrequency catheter ablation than those with AF lasting for 24 h or longer (6). Electrical and structural remodeling changes of the atrium, which occurred in 24 h and achieved a steady-state as early as 48 h after the onset of an AF episode, played an important role in increasing atrial vulnerability to AF induction and duration (13–15). This parallels the observational findings of substantial decreases in the conversion of AF to sinus rhythm after 24 continuous hours of AF onset.

### Recurrence of Atrial Tachyarrhythmia

As previously reported, AADs therapy for patients with AF had obviously high symptomatic AF recurrence compared with cryoablation (2). Cryoablation of AF, aiming for circumferential lesions and durable electrical isolation of the PVs, was non-inferior to radiofrequency-based ablation in terms of efficacy and safety for the treatment of patients with symptomatic paroxysmal AF, for whom at least one antiarrhythmic drug had failed (16, 17). However, in the FIRE and ICE study, a significant proportion of patients underwent first-generation cryoballoon ablation. Additionally, the second-generation cryoballoon ablation, facilitated shorter time to PVs isolation procedure and enhanced higher rates of freedom from AF, was considered as a reasonable and promising choice for patients suffering from paroxysmal AF (9, 18). The STOP AF trial demonstrated that cryoablation with the second-generation cryoballoon is an effective alternative to antiarrhythmic treatment of patients with symptomatic and drug-refractory AF (2). Likewise, a large, prospective, randomized, and controlled study showed comparable efficacy of open irrigated radiofrequency and cryoballoon catheter ablation for PVs isolation in patients with paroxysmal AF (19). As in our study, the ablation technique was used with the second-generation cryoballoon.

Structural remodeling of the atrium due to myolysis, cardiomyocyte apoptosis, and the activation of fibrotic pathways *via* fibroblasts, is closely related to the pathophysiologic process underlying atrial arrhythmia (20). Also, AF-related electrical and structural remodeling began as early as 24 h after the occurrence of AF (13–15). Evolving evidence showed that earlier catheter ablation may be a useful strategy in AF rhythm-reversion and maintenance of sinus rhythm, thus improving the prognosis of AF (21). The CIRCA-DOSE study about the early ablation of AF, with lesser recurrences of AF and lower post-ablation AF burdens compared with ablation of AF longer than 24 h, led to the notion that early cryoablation of AF was a beneficial tool in preventing AF development (6). However, these previous studies had not adequately assessed the effects of AF episodes of <24-h duration on the recurrence of atrial tachyarrhythmia after cryoablation. In our study, only a 24-h time point was selected to assess the impact of AF episodes duration on clinical outcomes and prognosis after cryoablation. Two different ablation types, including RF ablation and cryoablation, mixed together to perform secondary analysis of the CIRCA-DOSE study, may lead to information bias and selection bias (6).

In the current study, we confirmed that cryoballoon catheter ablation, an ablation technique for PVs isolation in patients with paroxysmal AF, had lower AF recurrences after a blanking period following cryoablation in consistent with prior studies. Furthermore, the present study showed evidence that cryoablation for paroxysmal AF lasting for 24 h or longer conferred a significantly greater recurrence of atrial tachyarrhythmia after ablation as compared with cryoablation for <24-h AF, especially in 4–9 months follow-up period. Early AF recurrence was negatively correlated with extensive PV reconnections after the blanking period (22). This observation is in consistent with the idea that early intervention, including radiofrequency and cryoballoon catheter ablation for PVs isolation, for paroxysmal AF episodes of <24-h duration, is acknowledged as a cutoff time point of the onset of the progressive anatomic and electrical changes related with atrial arrhythmia, may improve clinical prognosis.

### Adverse Clinical Events

AF cryoablation offers several potential advantages over AADs therapy for AF, such as arrhythmia freedom and safety outcomes (2). The FIRE and ICE trial demonstrated that there was no significant difference between the cryoballoon and radiofrequency ablation for paroxysmal AF with regard to overall safety (17). Cryoballoon ablation seems to be associated with a significantly lower risk of a serious complication in comparison to RF ablation, such as pericardial effusion and tamponade (23). Successful catheter ablation of AF reduced the risk of the total vascular events in patients with AF with CHA_2_DS_2_-VASc score ≥ 1, including stroke/TIA (24). A previous study showed that phrenic nerve palsies were more frequent in patients with AF by second-generation cryoballoon ablation than patients with AF by RF ablation, and recovered in all patients during the follow-up period (18). The overall incidence of atrial esophageal fistula remains rare in both RF and cryoballoon ablation procedures (23).

The CIRCA-DOSE study had not assessed the effects of AF recurrence after ablation on the clinical outcome of stroke/TIA (6). Higher incidence of stroke/TIA was investigated in patients with paroxysmal AF lasting for 24 h or longer compared with patients with AF episodes of <24-h duration in our study after cryoablation. AF recurrence is an independent risk factor for stroke/TIA. The possible explanations may be AF recurrence and delayed oral anticoagulant drugs administration after cryoablation in patients with paroxysmal AF lasting for 24 h or longer. Successful cryoablation reduced the risk of stroke/TIA in patients with AF episodes of <24-h duration. Likewise, in a previous study, the risk of ischemic stroke has been observed to increase substantially among patients with AF lasting for 24 h or longer (25, 26). High incidence of stroke/TIA was investigated in patients with AF lasting for 24 h or longer, which led to the notion that AF with >24 h was a highly relevant threshold for oral anticoagulation initiation.

The safety of early cryoablation was not evaluated in the CIRCA-DOSE study previously (6). In the current study, no significant difference in peri-procedural complications was observed between the groups. A procedural complication, PNI, was induced and maintained due to freezing-induced large axonal loss of phrenic nerve during the cryoablation step. However, the incidence rate of PNI was low in the whole patient, mostly recovered from diaphragmatic palsy during the follow-up period. Moreover, the risk for MACE, including all-cause death and cardiac death, was comparative in patients with AF episodes of <24-h duration with that in patients with paroxysmal AF lasting for 24 h or longer during the follow-up period. In general, our study revealed that cryoablation for treating paroxysmal AF episodes of <24-h duration is a safe tool for the first-line treatment of symptomatic AF.

## Limitations

There were several potential limitations to this study. First, it was a single-center and retrospective study, which was not designed in advance before the cardiac events occurred. Further prospective and randomized trials are required to confirm whether cryoablation for treating AF episodes of <24-h duration is effective and safe as compared with paroxysmal AF lasting for 24 h or longer. Second, only 253 enrolled patients were matched on a 1:1 basis by using identical propensity scores in this study. Therefore, there may be some information bias. Third, as the death rate is low in these cryoablation patients of our study, a further study with much larger sample size and long-term follow-up is necessary to get more exact data information about the mortal difference of patients with AF. Fourth, asymptomatic or short-lasting AF episodes may occur unnoticed by symptoms of the patients, ECG, and scheduled 24-h Holter monitoring. Therefore, there may be some overestimated effectiveness of the results. Finally, a potential mechanism of the occurrence and maintenance of AF, fibrosis of atrial myocytes, was not analyzed among the whole population in this study.

## Conclusion

In conclusion, patients with <24-h AF episodes are at significantly reduced risks of stroke/TIA and recurrence of atrial tachyarrhythmia when compared to patients with paroxysmal AF lasting for 24 h or longer. In this study, <24-h paroxysmal AF type and >40 mm LA diameter were independently related to the incidence of recurrence of atrial tachyarrhythmia.

## Data Availability Statement

The original contributions presented in the study are included in the article/supplementary material, further inquiries can be directed to the corresponding authors.

## Ethics Statement

This study was granted an exemption from requiring ethics approval by Ethics Committee of Shanghai Tenth People's Hospital because it was a retrospective observational study. The patients/participants provided their written informed consent to participate in this study.

## Author Contributions

CJ conceived and designed this study, performed cyroablation procedures, analyzed the data, and wrote the manuscript. DZ and KT carried out subjects recruitment and data interpretation and performed cryoablation procedures. YW, XL, and PJ contributed to data collection and manuscript revision. YX and BH contributed to the supervision of this study. All authors contributed to the article and approved the submitted version.

## Conflict of Interest

The authors declare that the research was conducted in the absence of any commercial or financial relationships that could be construed as a potential conflict of interest.

## Publisher's Note

All claims expressed in this article are solely those of the authors and do not necessarily represent those of their affiliated organizations, or those of the publisher, the editors and the reviewers. Any product that may be evaluated in this article, or claim that may be made by its manufacturer, is not guaranteed or endorsed by the publisher.
